# Performance assessment to improve public health systems

**DOI:** 10.2471/BLT.24.291543

**Published:** 2024-05-08

**Authors:** Jochen O Mierau, Simon van der Pol, Amrit Sandhu, Danielle EMC Jansen

**Affiliations:** a Department of Economics, Econometrics and Finance, Faculty of Economics and Business, University of Groningen, Nettelbosje 2, 9747 AE Groningen, Kingdom of the Netherlands.; b Health-Ecore, Zeist, Kingdom of the Netherlands.; c University Medical Centre Groningen, Groningen, Kingdom of the Netherlands.

Public health systems are under pressure, especially from the increase in noncommunicable diseases, rising health disparities as well as the coronavirus disease 2019 (COVID-19) pandemic.^1^ As unresolved health issues eventually put a burden on the health system, preventing disease is clearly a better approach than curing it. Moreover, while health at the individual and population level holds intrinsic value, it is also indispensable for proper societal and economic functioning. Health is a key production factor that needs to be valued in the same way as human and financial capital.^2^ However, while scientists, policy-makers and health workers acknowledge that prevention is better than cure, the dominant focus of health systems is on curing disease,^3^ as evidenced, for instance, by the small share of health expenses allocated to prevention.

Health systems are insufficiently equipped to deal with the pressure. Hence, focusing on strengthening public health systems through concerted actions by citizens, policy-makers and the global health community is needed. A key tool in achieving this objective is health system performance assessment, as it provides a systematic method to uncover the strengths and weaknesses of the health system.

In this article, we outline the potential and challenges of applying the Health System Performance Assessment Framework for Universal Health Coverage to public health systems.^4^ We start with conceptual issues, then showcase how a version of the assessment has been applied to assessing public health systems in nine jurisdictions, and conclude with a selection of lessons drawn from the study as well as on ways in which the assessment can be made more applicable to public health systems.

The assessment is a new framework that illustrates the relationship between the performance of health system functions and the intermediate objectives and final goals of the health system.^4^ The health system functions are: governance, resource generation, financing and service delivery, where service delivery is split into three components: public health, primary health care and specialized care.

While the position of public health within the assessment’s framework is clear, it struggles with the scope and boundaries of public health. Taking a common definition of public health as a starting point in the assessment (improving health, prolonging life and improving the quality of life among whole populations),^4^ it becomes clear that public health can be viewed narrowly, as the delivery of preventive services (such as vaccinations and health education) or broadly, as anything that promotes and protects health (such as labour laws and sewage systems). Moreover, differentiating public health from primary care is inherently difficult, as primary health care workers may be the ones who are delivering public health services.

The assessment clarifies that improving health is a central goal of any health system. However, large-scale improvements in health often stem from outside the health system.^5^ This challenge is compounded by the realization within the public health community that many contemporary impediments to public health stem from commercial interests.^6^ For example, tobacco and ultra-processed food, as well as the social media and gambling sectors, among many others, benefit from (over) consumption of health-harming goods or services. Once disease is present, pharmaceutical, medical technology and related firms benefit from treating avoidable diseases. Such diseases could, therefore, generate dual financial benefits for private parties. Public health policy that can robustly regulate and eventually eliminate these private business models would contribute to a societal business model, as healthier citizens are more productive both economically and socially.

In addition, climate change is increasingly putting a burden on public health around the world.^7^ Heat-related deaths of individuals aged 65 years and older have increased by about 85% between 2004 and 2022, which is more than twice the increase that was expected if temperatures had not increased.^7^ Yet the assessment does not consider measures taken to either mitigate climate change or adjust to it.

Therefore, having included public health in the assessment is positive as it relates to many determinants of health outside the health-care system, but the breadth of its definition is insufficiently complete. Indeed, if it is defined too narrowly, some of the largest contributions to health improvement such as sanitation and food safety are left out of scope. However, if the public health system is defined too broadly, an assessment of its performance becomes increasingly difficult.

## Application of the assessment 

A recent study provides a first application of the public health system performance assessment in nine jurisdictions (Australia, British Columbia [Canada], Chile, Denmark, England [United Kingdom of Great Britain and Northern Ireland], Italy, Latvia, Kingdom of the Netherlands and Singapore).^8^ These jurisdictions were selected based on guidance from the European Observatory on Health Systems and Policies, which involved consulting experts in health systems as well as looking at geographical spread and health-care system characteristics. Moreover, because the lessons drawn from the assessment were aimed at supporting advice on strengthening the Dutch public health system, the jurisdictions needed to be comparable to the Dutch system.^9^ One of the study’s shortcomings is that only relatively high-income countries were covered – and therefore their public health challenges may not reflect those of low- and middle-income countries.

The researchers took a broader view of public health than that in the assessment, in the sense that public health was not seen only as a means of service delivery. The researchers also considered the governance, resource generation, financing and service delivery of the public health system. To limit the scope of the study, and considering available resources and time, six tracer themes were identified as focal points of the assessment: urban planning, particulate matter disposal, human-papillomavirus vaccination, influenza management, mental health services and child screening services.

Between November 2021 and March 2022, two health system experts for each of the nine jurisdictions completed a questionnaire. Before distributing the questionnaires, a scoping review of scientific and grey literature was performed to pre-fill the questionnaires, allowing the local health system experts to focus on reviewing large parts of the questionnaire instead of completing the entire questionnaire. The researchers supplemented the information from the scoping review in combination with the input of the experts with indicators from, among others, the World Bank, the World Health Organization and the Organisation for Co-operation and Economic Development. 

The outcome of the study highlighted the difficulty of providing a consistent scope of public health systems.^10^ Indeed, while most jurisdictions have stated public health goals, some countries have specific subgoals. Italy, for instance, has an explicit focus on food safety; while Chile, England, Latvia and Kingdom of the Netherlands have goals focused on decreasing health disparities; and Australia and British Columbia have specific goals regarding indigenous populations.

Moreover, the study revealed differences in governance structures as well as resource generation, financing and service delivery. The governance of the public health system is generally outlined in one or more public health acts, especially for larger jurisdictions’ responsibilities, which are often divided across multiple levels of government. An element of shared responsibilities and resources across different policy domains is also often present.

## Lessons learnt 

This article does not intend to detail the various findings; rather, it presents the two key lessons drawn from the assessment.

First, while many rules and regulations are in place to govern public health, politicians at the national, regional and local levels are not held accountable if health goals are not met. Health goals are aspirational but not targets such as those set for public finances (such as fiscal debt and deficit rules)^11^ or the environment (such as emission targets). Hence, a key strategy to strengthen public health systems is to assign accountability for policy actions as well as outcomes.^12^

Second, funding for prevention should be seen as an investment in future health and not as a budgeted expense. Financing of public health is inherently different from health care, since usually medical services are budgeted to reduce short-term increases in costs as much as possible. However, doing so is counterproductive for preventive interventions as they are not primarily aimed at reducing costs but at enhancing health.^3^

## The way forward

In this article, we have outlined the role that performance assessment can play in strengthening public health systems by providing a systematic method of assessing the strengths and weaknesses of public health systems. In doing so, we have also suggested some directions towards which the assessment can be developed further. Indeed, scholars applying the assessment will have to consider whether the place of public health in the current assessment, as a means towards service delivery, properly reflects the broad role that public health policy can potentially play in improving health and reducing disparities. Embedding the interaction between public health and commercial determinants of health, as well as climate change, more closely in the assessment will also be important. While lessons could be drawn from the nine jurisdictions in which the assessment was applied – especially regarding accountability and the investment nature of prevention – moving forward, pursuing an assessment of public health systems across a much broader range of jurisdictions will be needed. Although doing so poses many difficulties, such broader application can contribute to strengthening public health systems, considering the challenges posed by communicable and noncommunicable diseases as well as the large socioeconomic health disparities worldwide.
